# Comparison of clinical and virological features in pediatric and adult dengue cases at Insein General Hospital during Myanmar’s 2022 dengue season

**DOI:** 10.1186/s41182-025-00688-7

**Published:** 2025-01-29

**Authors:** Htin Lin, Mya Myat Ngwe Tun, Yin Mon Wint Zin, Khin Theingi Myint, Win Kay Khine, Khine Mya Nwe, Aye Aye Thant, Thin Thin Shwe, Win Mar, Khin Sandar Aye, Khaing Moe Aung, Yuki Takamatsu, Wah Wah Aung, Yi Yi Kyaw, Takeshi Urano, Kouichi Morita, Hlaing Myat Thu

**Affiliations:** 1 Department of Medical Research, Ministry of Health, No.5, Ziwaka Road, Dagon Township, Yangon, 11191 Myanmar; 2 Department of Virology, Institute of Tropical Medicine, Nagasaki University, 1-12-4 Sakamoto, Nagasaki, 852-8523 Japan; 3 Department of Tropical Viral Vaccine Development, Institute of Tropical Medicine, Nagasaki University, 1-12-4 Sakamoto, Nagasaki, 852-8523 Japan; 4 Center for Vaccines and Therapeutic Antibodies for Emerging Infectious Diseases, Shimane University, 89-1 Enya-Cho, Izumo, 693-8501 Japan; 5 Insein Teaching and General Hospital, Yangon, Myanmar; 6 Dejima Infectious Diseases Research Alliance, Nagasaki University, Nagasaki, Japan

**Keywords:** Dengue, Serotype, Myanmar, Children, Adult

## Abstract

**Background:**

Myanmar is one of the countries in Southeast Asia where serious dengue outbreaks occur and Yangon is among the regions with the highest number of cases in the country. Many infections including dengue are common in Yangon during the rainy season, and co-infections may also occur. Adults are more likely than children to experience co-infections of dengue and other diseases. Although pediatric dengue has been studied in Yangon for decades, research on adult dengue is scant. Therefore, this study compared the clinical and virological characteristics of pediatric and adult dengue cases in Yangon.

**Methods:**

This cross-sectional study was conducted at Insein General Hospital in Yangon, Myanmar, from June to September 2022. We recruited 221 suspected dengue patients (134 children and 87 adults), with or without other diseases, and tested their dengue serological markers using a serological method and their dengue virus (DENV) serotypes using conventional RT-PCR. Chi-squared and Fisher’s exact tests were conducted to assess significance.

**Results:**

The dengue non-structural protein-1 antigen (NS1Ag) positivity was 37% in children and 32% in adults. DENV serotypes were identified in 80% of NS1Ag-positive patients. Among NS1Ag-positive cases, the DENV-1 serotype predominated (67%), followed by DENV-2 (17%), DENV-3 (9%), DENV-4 (5%), and mixed DENV-1 and DENV-2 (2%) serotypes. Shock was observed in 14% of children and 3% of adults. Anti-dengue IgG antibody positivity was positively correlated with dengue shock. Three pediatric dengue cases (6%) also had other infections including bronchiolitis, ear infection, and diarrhea. Seven adult dengue cases (25%) also had other diseases including advanced HIV infection, severe pneumonia, tonsillitis, thyroid disease, cholecystitis, drug poisoning, and thalassemia.

**Conclusion:**

The serotype distribution and clinical presentations of pediatric and adult dengue cases were not significantly different, but adults were more likely to have dengue together with other diseases than children. This study provides information for the better management of febrile children and adults in hospital settings and provides a foundation for nationwide epidemiological studies on dengue serotypes and modifications of the national guidelines for dengue management in Myanmar.

**Supplementary Information:**

The online version contains supplementary material available at 10.1186/s41182-025-00688-7.

## Introduction

Dengue has been reported in many regions of the world but 70% of the disease’s burden occurs in Asia. Dengue is a serious illness in the Americas, Southeast Asia, and the Western Pacific [1]. Over 7.6 million dengue cases were reported to the World Health Organization (WHO) by April 30, 2024, including 3.4 million confirmed cases, over 16,000 severe cases, and over 3000 deaths. Myanmar is a Southeast Asian country where dengue epidemics occur every 2 or 3 years. Documented cases of dengue fever in Myanmar go back to 1960. In 1970, Myanmar’s first documented dengue outbreak occurred in the Yangon Region and gradually spread to other states and regions. In 2015, all seven states and seven regions of Myanmar were affected by dengue. Yangon is one of the three regions with the highest number of dengue cases in the country. Dengue was previously recognized as a pediatric disease in Yangon; however, many adult dengue cases have been reported in the region since 2015, highlighting the importance of virological studies on both pediatric and adult patients [2].

Dengue, an arthropod-borne viral infection, is caused by dengue viruses (DENVs): DENV-1, DENV-2, DENV-3, and DENV-4. These viruses are mainly transmitted by *Aedes aegypti* and *Aedes albopictus* mosquitoes. Symptomatic dengue infection is associated with fever, lethargy, headaches, joint pain, eyeball pain, abdominal pain, and vomiting. Because these symptoms are non-specific, dengue patients may be misdiagnosed with other febrile illnesses, leading to late treatment and poor prognosis [1]. The clinical presentation and severity of dengue depend on the viral strain, reinfection, the patient’s age, underlying diseases like diabetes mellitus, and co-infections with other viruses including that causing coronavirus disease-19 (COVID-19) [3–5]. Co-infection of DENV and other pathogens including those causing chikungunya, Zika, influenza, COVID-19, malaria, typhoid, and rickettsiosis may result in more severe illness and misdiagnosis of dengue [5–10]. Many infections such as dengue, chikungunya, and influenza are prevalent in the rainy season in Myanmar [11–13]. Hence, dengue and other infections may co-exist in Yangon but there is scant evidence-based data. Apart from patients with infectious diseases, surgical patients may also suffer from dengue infections, leading to poor outcomes in the postoperative period if dengue is misdiagnosed [14]. There are many studies on the clinical characteristics of dengue in Myanmar but few studies have evaluated pre-existing diseases and co-morbidities in dengue patients in the country. Because adults are more likely to have underlying diseases than children, the co-existence of dengue with other diseases may occur more frequently in adults. However, the clinical characteristics (including co-morbidities) of dengue adult and pediatric patients in Myanmar should be compared to generate evidence-based data.

Insein General Hospital (IGH) is a tertiary public hospital in northern Yangon, Myanmar. The hospital has nine specialty inpatient wards including a child and a medical ward, where many dengue patients from Insein, Shwepyitha, Hlaingthaya, Taikkyi, and Hmawbi Townships are treated. Therefore, pediatric and adult dengue cases in the hospital were selected for a virological and clinical study. This study compared the virological and clinical characteristics of pediatric and adult dengue cases treated at IGH during the 2022 dengue season. The specific objectives were to compare the dengue positivity rate, predominant dengue serotypes, clinical severity, and co-morbid conditions of the pediatric and adult dengue infected patients.

## Materials and methods

### Study design, period, population, and sample collection

This cross-sectional study was conducted at 500 bedded Insein General Hospital (IGH), located in Insein Township, Yangon, during the rainy season (June–September) of 2022. Yangon is among the regions with the highest number of reported dengue cases in Myanmar and dengue peaks during the rainy season [2, 11]. Patients from various townships in Yangon are treated at IGH during the rainy season because of dengue-like symptoms. The temperature and relative humidity of these townships are generally alike because all of them are geographically close and their average elevation from the sea level is also similar (approximately 15 m above sea level) [15]. The study population comprised 134 children (≤ 12 years) and 87 adult patients with clinically suspected dengue, with or without other diseases. According to the 2009 WHO [1] guidelines, a patient is considered to have clinically suspected dengue if they present high fever and at least two of the following symptoms or signs: headache, nausea, vomiting, pain (including muscle or joint pain), rash, evidence of fragile blood vessels (a positive tourniquet test), leukopenia (white blood cell count ≤ 5000 cells/mm^3^), thrombocytopenia (platelet count ≤ 150,000 cells/mm^3^), or erythrocytosis (hematocrit increased by 5–10%). All the children and adults treated at the outpatient department and inpatient wards of IGH from June to September 2022 were recruited for the study. Venous blood (2–3 mL) was taken from each patient and the serum was separated. The patients’ demographic and clinical data were collected from their medical records kept at IGH. The full medical record of each patient was thoroughly reviewed after the patient was discharged from the hospital.

### Dengue serological test

Patients’ sera separated from the blood samples were tested for dengue serological markers such as the dengue non-structural protein-1 antigen (NS1Ag) and dengue-specific immunoglobulin M (IgM) and immunoglobulin G (IgG) antibodies by immunochromatography (LumiQuick Dengue Duo Panel, Santa Clara, CA USA). Dengue serological tests were conducted at the Microbiology Department of IGH as part of the routine investigations. Positive and negative control samples of dengue NS1Ag, anti-dengue IgM, and IgG antibodies from a previous study [16] were tested alongside the patient samples to confirm the validity of the testing procedure.

After informed consent was obtained, the leftover sera were transported to the Department of Medical Research (DMR), using cool containers for dengue serotyping.

### Viral RNA extraction and DENV serotyping

Sera positive for dengue NS1Ag were processed for dengue serotyping. Viral RNA was extracted from the sera using a QIAamp Viral RNA Kit (Qiagen, Hilden, Germany), according to the manufacturer’s guidelines. The presence of DENV RNA was verified by conventional reverse transcription-polymerase chain reaction (RT-PCR), using a QIAGEN OneStep RT-PCR Kit (Qiagen) along with dengue serotype-specific primers. DNase/RNase-free water (Sigma, New York, NY, USA) was used as a negative control, and the four serotypes of DENV from a previous study [16] were used as positive controls. The thermal conditions for four serotypes of dengue RT-PCR were as follows: reverse transcription at 50 °C for 45 min and initial denaturing at 95 °C for 10 min, 40 cycles of denaturing at 94 °C for 45 s, annealing at 54 °C for 45 s, extension at 72 °C for 1 min, and final extension at 72 °C for 10 min. The amplicons were mixed with DNA loading dye (Invitrogen, USA), loaded into 2% agarose gel, and electrophoresed at 100 V for 45 min. The images of the gel were analyzed using a gel electrophoresis system (Bio-Rad, USA). The reactive bands of 490, 230, 320, and 399 bp indicated the gene segments of DENV-1, DENV-2, DENV-3, and DENV-4, respectively. The reaction mixture for each PCR protocol contained 5 µL of sample RNA, 12 µL of deionized water, 5 µL of 5X buffer, 1 µL of 10 mM dNTP mixture, 0.5 µL of 100 pmol forward and reverse primers, and 1 µL of enzyme mixture with separate primer sets (Supplementary Table 1). These mixtures were used to detect DENV and determine the specific DENV serotype [17]. Supplementary Fig. 1 shows a representative DENV-RT-PCR gel image displaying all four serotypes.

### Operational definitions

Based on the WHO criteria [1], laboratory-confirmed DENV infection was defined through positivity on at least one of the molecular methods (virus isolation confirmed by a conventional RT-PCR test and/or viral genome detection from serum using a quantitative RT-PCR test) or serology tests (NS1 Ag detection by an immunochromatographic test kit or anti-DENV IgM antibody detection by an IgM ELISA test). Cases with anti-DENV IgM but without NS1 antigen in a single serum were not regarded as dengue infections in this study.

### Statistical analysis

SPSS version 16 (IBM, Armonk, NY, USA) was used for data analysis. Only the data of children aged ≥ 5 years were used to analyze pain symptoms in pediatric cases. Depending on the frequency of the categorical variable, Pearson’s chi-squared or Fisher’s exact tests were used to determine significant differences regarding the presence or absence of each symptom in pediatric and adult patients, the association between the anti-DENV IgG status of patients and with or without dengue shock, and the association between the patient’s age group and dengue severity; *P* < 0.05 was considered statistically significant.

## Results

### Profiles of NS1 Ag, IgM, and IgG in clinically suspected dengue cases

**Fig. 1:**
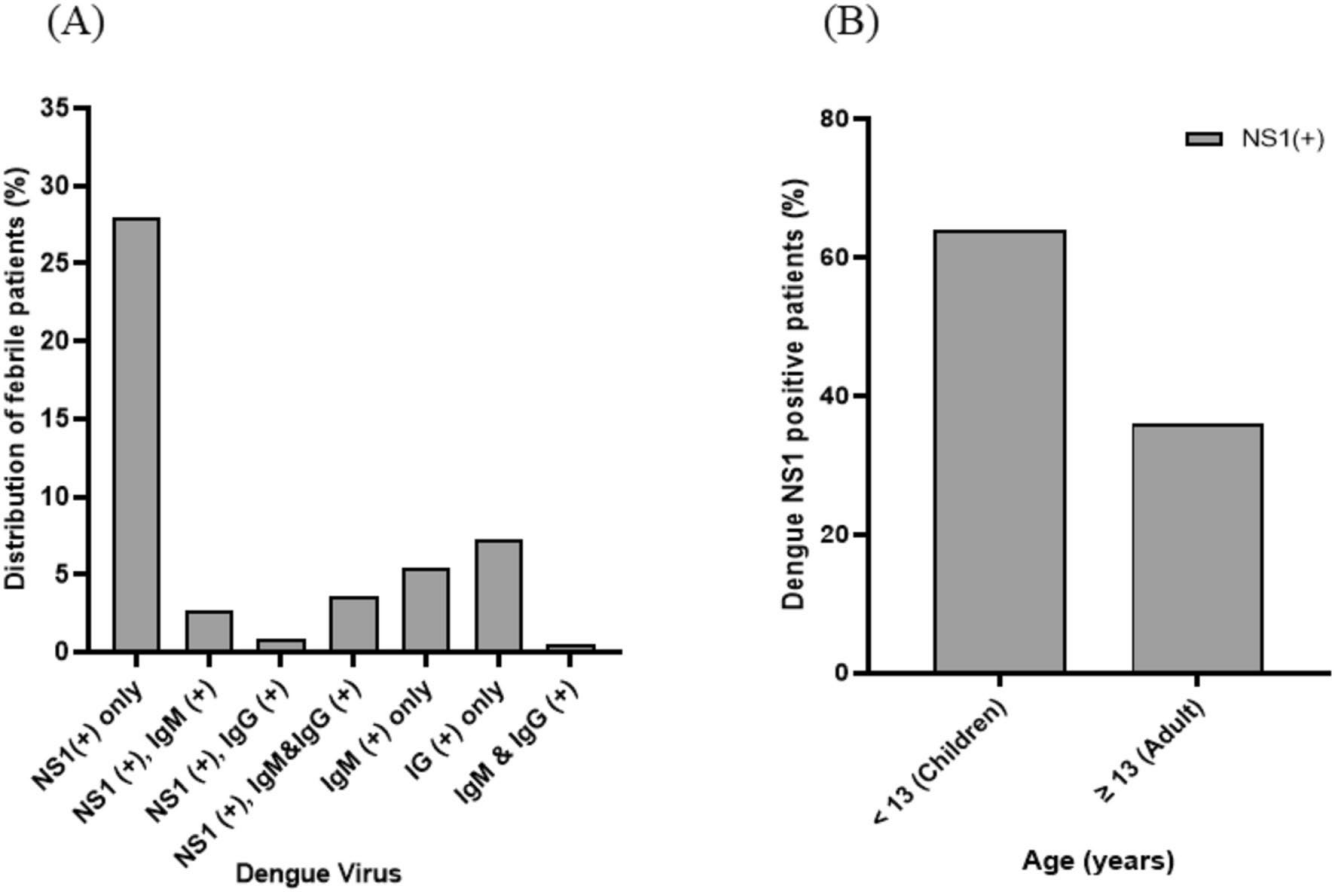
**A** Serological profiles and **B** NS1-positive cases in pediatric and adult patients at Insein General Hospital, 2022

In Myanmar, patients aged less than 13 years are treated as pediatric cases and those aged 13 years or more are treated as adult cases in hospital settings. In this study, of 221 patients (134 children aged 1–12 years and 87 adults aged 13–54 years) with clinically suspected dengue, 62 (28%), including a pregnant woman, were positive for dengue NS1 antigen alone, six (3%) were positive for NS1 antigen and anti-dengue IgM, two (0.9%) were positive for NS1 and anti-dengue IgG, eight (4%) were positive for NS1 and anti-dengue IgM and IgG, 12 (5%) were positive for anti-dengue IgM alone, one (0.5%) was positive for anti-dengue IgM and IgG, and 16 (7%) were positive for anti-dengue IgG alone. Based on the case definition of confirmed dengue, 78 patients (35%) with NS1 were regarded as dengue-positive cases (Fig. 1A).

### Characteristics of dengue-positive patients

The 37% (n = 50/134) of children and 32% (n = 28/87) of adults with clinically suspected dengue were positive for dengue NS1 antigen. The positivity rate was not significantly different between these groups (*P* = 0.43) (Fig. 1B). Among the 50 dengue-positive children, 27 (54%) were females and 23 (46%) were males. Among dengue-positive adults, 13 (46%) were females and 15 (54%) were males. In the pediatric group, females had a higher positivity rate than males but the difference was not significant (*P* = 0.16). In the adult group, males had a higher positivity rate than females but the difference was not significant (*P* = 0.59). The youngest dengue patient was aged 6 months and the oldest was aged 55 years. Patients aged less than 13 years had a higher positivity of DENV than those aged ≥ 13 years. However, the difference between the age groups was not significant (*P* = 0.61) (Fig. 1B).

### Distribution of DENV serotypes among children and adults

**Fig. 2:**
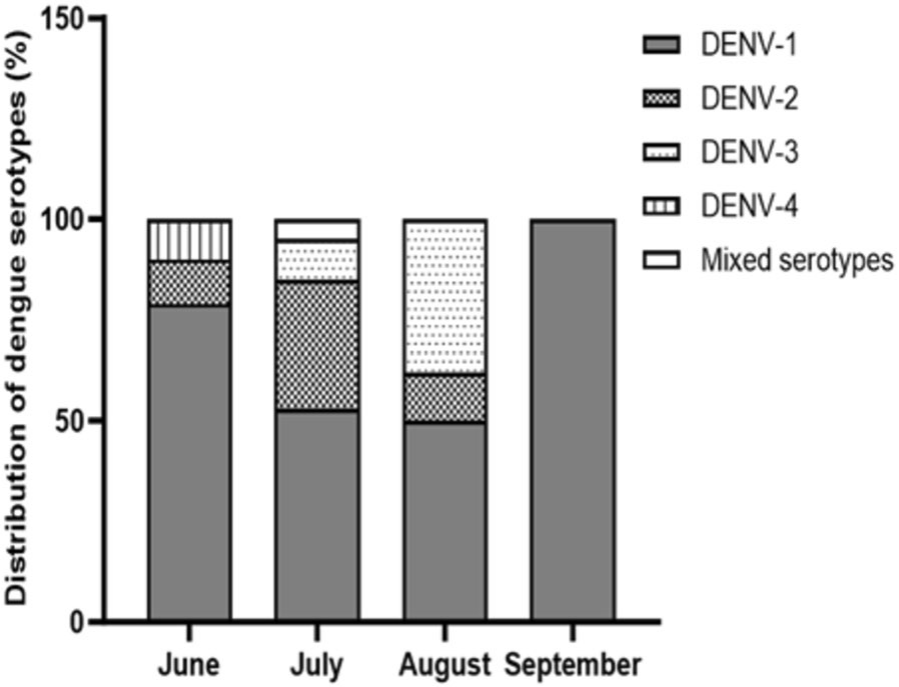
Monthly distribution of dengue serotypes which detected in pediatric and adult patients at Insein General Hospital, Myanmar in 2022

Among the 78 dengue NS1-positive cases, dengue serotypes were identified in 58 cases (74%). DENV-1 predominated in both children (40) and adults (18) and accounted for 27 (68%) and 12 (67%) cases, respectively, followed by DENV-2, which accounted for seven (18%) and three (17%) cases, respectively. DENV-3 and DENV-4 were detected in three (8%) and two (5%) dengue-positive children and two (11%) and one (6%) dengue-positive adults, respectively. Only one mixed type (DENV-1 + DENV-2) was detected among dengue-positive children (Table 1). In summary, DENV-1 (67%) predominated among both children and adults, followed by DENV-2 (17%), DENV-3 (9%), DENV-4 (5%), and a co-infection of DENV-1 and DENV-2 (2%). Thus, the frequency distribution of DENV serotypes did not differ between the pediatric and adult groups (*P* = 0.95) In June, DENV-1 was dominant (79%), followed by DENV-2 (11%) and DENV-4 (10%). In July, the positivity of DENV-1 declined to 53%, but that of DENV-2 increased to 32%, and that of DENV-3, to 10%. In July, the mixed serotype of DENV-1 and DENV-2 was detected in 5% of patients. In August, the positivity of DENV-1 dropped to 50%, and that of DENV-2, to 12%, whereas the positivity of DENV-3 increased to 38%. All dengue serotypes detected in September were DENV-1 (Fig. 2). Thus, DENV-1 was the predominant serotype throughout the dengue peak season and the maximum number of DENV-2 cases occurred in July (Fig. 2).

**Table 1:** Distribution of dengue virus serotypes among dengue NS1 antigen positive pediatric and adult patients at Insein General Hospital (IGH) in 2022

	Children		Adults		Both children and adults	
	Number of cases	%	Number of cases	%	Number of cases	%
DENV-1	27	68	12	67	39	67
DENV-2	7	18	3	17	10	17
DENV-3	3	8	2	11	5	9
DENV-4	2	5	1	6	3	5
DENV-1 + DENV-2	1	3	0	0	1	2
Total	40	100	18	100	58	100

### Clinical features of children and adult dengue-positive cases

Fifty dengue NS1-positive pediatric patients presented fever (50, 100%), vomiting (17, 34%) abdominal pain (15, 30%), bleeding manifestations (17, 34%), and maculopapular rash (11, 22%). On the other hand, 28 dengue NS1-positive adults presented fever (28, 100%), vomiting (7, 25%), bleeding manifestations (6, 21%), and maculopapular rash (7, 25%). The rates of vomiting and bleeding manifestations in children were higher than those in adults but the association was not statistically significant. Joint or muscle pain was more common in adults than children (≥ 5 years old), but the association was not statistically significant. Headaches occurred in two (4%) of 50 pediatric cases and four (14%) of 28 adult cases (Table 2).

**Table 2:** Clinical characteristics of children and adult dengue-positive cases at IGH in 2022

No	Clinical feature	Children dengue	Adult dengue	Total	*P*-value*
1	Fever				
	Present	50 (100%)	28 (100%)	78 (100%)	NA
	Absent	0 (0%)	0 (0%)	0 (0%)	
2	Vomiting				
	Present	17 (34%)	7 (25%)	24 (31%)	0.45
	Absent	33 (66%)	21 (75%)	54 (69%)	
3	Abdominal pain				
	Present	15 (30%)	6 (21%)	21 (27%)	0.29
	Absent	35 (70%)	22 (79%)	57 (73%)	
4	Bleeding manifest*ations*				
	Present	17 (34%)	6 (21%)	23 (29%)	0.30
	Absent	33 (66%)	22 (79%)	55 (71%)	
5	Maculopapular rash				
	Present	11 (22%)	7 (25%)	18 (23%)	0.78
	Absent	39 (78%)	21 (75%)	60 (77%)	
6	Joint/muscle pain				
	Present	2 (4%)	6 (21%)	8 (10%)	0.13
	Absent	48 (96%)	22 (79%)	70 (90%)	
7	Headache				
	Present	2 (4%)	4 (14%)	6 (8%)	0.18
	Absent	48 (96%)	24 (86%)	72 (92%)	
8	Cough				
	Present	4 (8%)	2 (7%)	6 (8%)	1
	Absent	46 (92%)	26 (93%)	72 (92%)	
9	Febrile convulsion				
	Present	5 (10%)	0 (0%)	5 (6%)	NA
	Absent	45 (90%)	28 (100%)	73 (94%)	
10	Diarrhea				
	Present	1 (2%)	2 (7%)	3 (4%)	0.59
	Absent	49 (98%)	26 (93%)	75 (96%)	
11	Shock				
	Present	6 (12%)	1 (4%)	7 (9%)	0.41
	Absent	44 (88%)	27 (96%)	71 (91%)	

^*^Calculated by Chi-square or Fisher’s exact test

Among NS1-positive patients, dengue shock was observed in six (12%) children and one (4%) adult. Febrile convulsion was detected in five (10%) children but not in adults. No significant association was observed between dengue shock and the patient’s age group (*P* = 0.70). However, dengue NS1-positive patients with anti-dengue IgG developed shock more frequently than those without anti-dengue IgG (*P* = 0.04) (Table 3).

**Table 3:** Association between the anti-dengue IgG status of patients and dengue shock

	Dengue shock	No dengue shock	Total	*P*-value*
Dengue NS1 positive, anti-dengue IgG positive	3 (30%)	7 (70%)	10 (100%)	0.0412
Dengue NS1 positive, anti-dengue IgG negative	4 (6%)	64 (94%)	68 (100%)	
Total	7 (9%)	71 (91%)	78 (100%)	

^*^Calculated by Chi-square or Fisher’s exact test

In the pediatric group, most of the dengue cases 29 (58%) had no warning signs, 15 (30%) had warning signs, and six (12%) developed dengue shock. Such distribution of cases was also seen in the adult group. Therefore, there was no association between the patient’s age group and the severity of dengue (*P* = 0.34) (Table 4). When the clinical severity of dengue was compared with the DENV serotypes, DENV-1 was more likely to result in dengue without warning signs or shock than DENV-2, DENV-3, and DENV-4 (Table 5). The patients with dengue shock were infected with DENV-1, DENV-2, and DENV-4.

**Table 4:** Association between the age group of the patients and dengue severity

	Without warning sign	With waring sign	Severe dengue (shock)	Total	P-value*
Pediatric Dengue	29 (58%)	15 (30%)	6 (12%)	50 (100%)	0.34
Adult Dengue	19 (68%)	8 (29%)	1 (3%)	28 (100%)	
Total	48 (62%)	23 (29%)	7 (9%)	78 (100%)	

^*^Calculated by Chi-square or Fisher’s exact test

**Table 5:** Association between severity of dengue and dengue serotypes

	Dengue without warning signs	Dengue with warning signs	Dengue shock
DENV-1	25 (71%)	10 (59%)	4 (67%)
DENV-2	6 (17%)	3 (18%)	1 (17%)
DENV-3	2 (6%)	3 (18%)	0 (0%)
DENV-4	1 (3%)	1 (6%)	1 (17%)
DENV-1 + DENV-2	1 (3%)	0 0%)	0 (0%)
Total	35 (100%)	17 (100%)	6 (100%)

### Co-morbidities or complications in dengue-positive patients

**Fig. 3:**
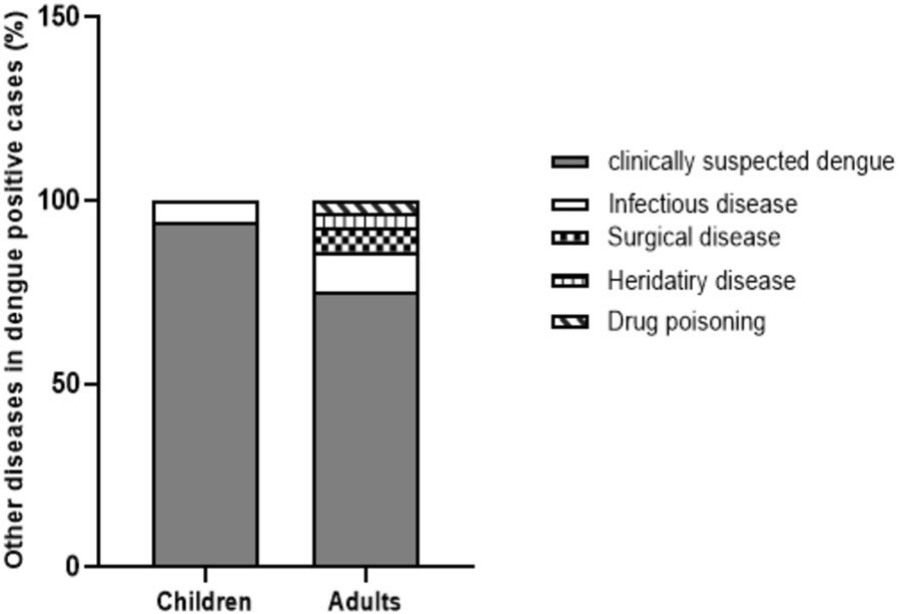
Frequency distribution of other diseases in pediatric and adult dengue patients treated at ITGH in 2022

Out of 50 pediatric dengue NS1-positive patients, 47 (94%) went to the hospital because of suggestive symptoms of dengue (SSD) including fever, nausea, vomiting, upper abdominal pain or discomfort, aching pain, or bleeding manifestations, but three (6%) were admitted for infectious diseases (bronchiolitis, ear infection, and acute gastroenteritis (acute watery diarrhea and vomiting)) although they also exhibited SSD and were infected with DENV-1. Among the 28 dengue NS1-positive adults, 21 patients (75%) went to the hospital with clinically suspected dengue, but seven (25%) went for other causes. Among the latter, three (10.7%) had infectious diseases (acute tonsillitis, severe pneumonia, and advanced human immunodeficiency virus (HIV) infection with syphilis), two (7.1%) suffered from surgical diseases (calculus cholecystitis and solitary thyroid nodule), one (3.6%) had a hereditary disease (anemia with beta-thalassemia), and another one (3.6%) had experienced drug poisoning (drug-induced poisoning with SSD). The drug-induced poisoning case had taken 10 tablets of vitamin B1, B6, and B12 1 day before admission to IGH (Fig. 3). Among the adult dengue-positive patients with co-morbidities, the four patients who had HIV, acute tonsillitis, solitary thyroid nodule, and drug poisoning were infected with DENV-1, whereas the two patients with anemia and calculus cholecystitis were infected with DENV-2. However, we could not identify the DENV serotype for the patient with severe pneumonia. In summary, DENV-1 infection was more prevalent in pediatric and adult patients who had co-morbidities.

## Discussion

Dengue NS1 Ag rapid tests have an overall specificity of 96–100% but anti-dengue IgM or IgG tests have specificities of 40–50% because DENV and other flaviviruses—especially the Zika virus—exhibit high structural similarity and cross-reactive immune response [11–14]. Thus, positive cases identified by anti-dengue antibodies are more likely to include false positives compared to those identified by the dengue NS1 Ag [12, 13, 18]. Moreover, a positive result for anti-dengue IgG may be due to either a previous dengue infection or a secondary dengue infection, and quantitative serological tests are required to distinguish between them [19, 20]. Here, acute single sera from pediatric and adult patients were available. If paired sera from patients could have been subjected to a quantitative serology test for dengue, the positivity rate in this study may have surpassed 35%.

Similar dengue positivity rates were found for the pediatric (37%) and adult (32%) groups in this study. A high prevalence of dengue in adolescents has been observed in Malaysia and Thailand [21, 22]. Although no sex preponderance was observed among pediatric and adult cases, clinical suspicion and prevention of dengue should focus on children and young adults of both sexes. The lack of significant age and sex differences in dengue positivity is clear. However, the high prevalence of dengue positivity among adolescents necessitates further exploration of social and environmental factors.

Dengue is one of the leading causes of morbidity and mortality among children under 15 years in Myanmar. Pediatric patients are more affected by dengue than adults likely because of immunological differences that make children more susceptible to the virus. The smaller body size and lighter weight could contribute to the severe symptoms experienced by children. Furthermore, their tendency to play outdoors increases their exposure to mosquitoes active during the daytime. On the other hand, most adults in Myanmar generally do not visit hospitals if they have only minor symptoms like fever and moderate pain. No nationwide investigation has been conducted to determine asymptomatic adult dengue cases in Myanmar. Therefore, determining DENV infection among asymptomatic adults is needed to clarify the impact of the disease in the country.

The frequency distribution of dengue serotypes in the study area was not significantly different for children and adults. We can assume that the circulating pattern of dengue serotypes does not depend on the age of people living in the same area. However, the circulating pattern of the disease may change geographically because, in 2017, DENV-1 predominated in Myanmar, whereas DENV-2 predominated in Bangladesh, and DENV-3, in Western India [23–25]. DENV-1 (68%) predominated in both children and adults in our study. Previous studies among children in Yangon found a predominance of DENV-1 in 2016, 2018, and 2020 [26]. According to the Vector Borne Disease Control Programme of Myanmar’s Ministry of Health, DENV-1 was the most common serotype in Myanmar during 1999–2022 [17, 23]. The long-term transmission of a defective lineage of DENV-1 in Myanmar was first noticed in 2001 [27], raising important questions about the emergence of transmissible defective viruses and their role in viral epidemiology. By combining genetic sequence analysis and mathematical modeling, the same group of researchers provided evidence of the transmission of a defective DENV, primarily through co-transmission with the functional virus to uninfected individuals. Surprisingly, this co-transmission route had a higher transmission potential than functional dengue viruses alone [28]. Thus, future studies should investigate the association between defective DENV-1 viruses and dengue outbreaks in Myanmar.

The positivity rate of DENV-2 was 18% in the pediatric group and 17% in the adult group in this study. Previous studies in Yangon did not find DENV-2 among children in hospitals in Yangon during 2016–2019. However, in 2020, DENV-2 was detected in 25% of dengue-affected children in hospitals in Yangon. DENV-2 is associated with severe dengue and shock; thus, dengue serotyping should be continuously performed for the early recognition of this serotype [29]. DENV-3 and DENV-4 were the dominant serotypes among children in Yangon in 2017 and 2019, respectively [30], but were less prevalent in 2022. On the other hand, DENV-1, which was less prevalent in Yangon in 2017 and 2019 [31], was dominant in 2020 and 2022, highlighting the changing pattern of DENV serotypes in Yangon. These findings highlight the importance of continuously monitoring dengue serotypes in Yangon to predict and prepare for dengue outbreaks in the region.

During the study period, the maximum number of DENV-1 cases was observed in June. The positivity rate of DENV-2 increased in July, and that of DENV-3, in August. These findings indicate that a shift in the dengue serotype may occur even in the peak season of the disease. Thus, the predominant dengue serotype should be determined monthly throughout the entire dengue season.

A study in Brazil found a significant association between arthralgia and the age of dengue patients but the association was not significant in the current study [32]. This may be due to the small number of adult dengue cases in the study population. Fever and arthralgia are non-specific symptoms of dengue, and they also occur in many diseases including influenza, chikungunya, and COVID-19 [5, 33, 34]. Furthermore, there have been documented cases of dengue co-infections with these viruses [35, 36].

Shock was not significantly associated with the patient’s age group but was associated with the patient’s anti-dengue IgG status, which demonstrates the crucial role of dengue serological markers in predicting dengue shock. Some studies have found an association between dengue serotypes and dengue shock. However, that was not the case in our study. Nonetheless, the limited number of dengue shock cases may have affected our results. Apart from the positivity of anti-dengue IgG, many factors may influence the risk of developing dengue shock, including the DENV genotypes, the level of cytokines such as IL-6, IL-8, and anti-dengue IgM in the patient’s serum, and the primary subclass of anti-dengue IgG (anti-dengue IgG1, IgG2, IgG3, and IgG4) that developed in the patients during the course of the disease [37–40].

Three pediatric dengue cases co-existed with other infections like bronchiolitis, acute gastroenteritis, and ear infection with discharge. Bronchiolitis and acute gastroenteritis may be either dengue co-infections or complications [41, 42]. Thus, dengue should be suspected in every febrile child presenting common or uncommon symptoms of the disease. An adult dengue patient who was treated at IGH presented fever, vomiting, and abdominal pain and was initially diagnosed as having calculus cholecystitis by an abdominal ultrasonographic scan, followed by the serological diagnosis of dengue. Therefore, febrile patients with medical diseases and those with surgical diseases should be tested for dengue regardless of a confirmed diagnosis of other diseases.

In addition, dengue was detected in adults with non-communicable diseases such as thalassemia and drug poisoning presenting dengue-like symptoms such as fever, lethargy, and a positive tourniquet test. A triple infection with DENV, HIV, and *Treponema pallidum* was also detected in this study but such cases are rare. However, dual infections with DENV and HIV have been occasionally reported [43, 44]. Therefore, dengue should be considered when testing febrile patients for HIV, especially during the dengue season. Some co-infections of influenza/dengue cause progressive lung damage [45], whereas some co-infections of HIV/DENV result in a benign clinical progression of dengue [46]. Thus, considering dengue in every febrile case can reduce misdiagnosis of the disease in co-infection cases or dengue cases mimicking other diseases. Moreover, it can prevent unnecessary interventions and reduce hospital stays and hospitalization expenditures.

Most (70%) dengue co-infections had DENV-1 as the predominant strain during the study period. However, because of the small number of co-infections, an association between the dengue serotype and the co-infection with other pathogens could not be determined. Because Myanmar is a dengue-endemic country, any serotype of dengue dominant in a year may co-exist with other diseases in the same year, especially during the rainy season.

This study faced some limitations. Dengue serotypes could not be identified in 20% of serologically confirmed dengue cases. As previously mentioned, this may be due to the low viral load [47] or point mutations [48]. Moreover, the small number of dengue shock cases with identifiable serotypes affected the significance of the association between these variables. The small number of dengue co-infections with other pathogens also made an association with dengue serotypes difficult.

In conclusion, the serotype distribution and clinical presentations of pediatric and adult dengue cases were not significantly different. However, dengue and other diseases were more likely to co-exist in adults than in children. This study provides information for the better management of febrile children and adults in hospital settings and may serve as the basis for further nationwide epidemiological studies on dengue serotypes and the modification of national guidelines for dengue management in Myanmar.

## Supplementary Information

Supplementary Material 1.

Supplementary Material 2.

## Data Availability

No datasets were generated or analysed during the current study.

## Abbreviations

NS1: Non-structural protein 1
DENV: Dengue virus
DENVs: Dengue viruses
HIV: Human immunodeficient virus
WHO: World Health Organization
COVID 19: Coronavirus Disease-19
ITGH: Insein Teaching and General Hospital
IgM: Immunoglobulin M
IgG: Immunoglobulin G
RT-PCR: Reverse Transcriptase Polymerase Chain Reaction
SSD: Suggestive symptoms of dengue
