# Supplementary figures and images for: Comparison of clinical and virological features in pediatric and adult dengue cases at Insein General Hospital during Myanmar’s 2022 dengue season
Source: Trop Med Health. 2025 Jan 29;53:13. doi: 10.1186/s41182-025-00688-7 (PMC11780819; doi:10.1186/s41182-025-00688-7)

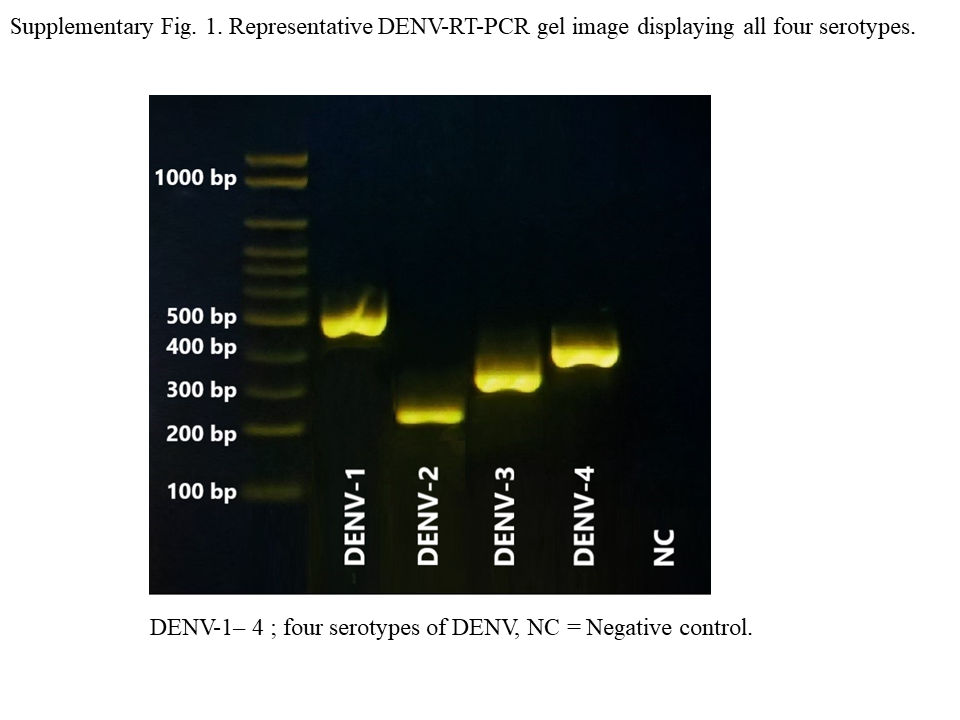

Supplement: Supplementary file 1 — Supplementary Material 1. [file 41182_2025_688_MOESM1_ESM.tif]
