# Supplementary material for: Comparison of clinical and virological features in pediatric and adult dengue cases at Insein General Hospital during Myanmar’s 2022 dengue season
Source: Trop Med Health. 2025 Jan 29;53:13. doi: 10.1186/s41182-025-00688-7 (PMC11780819; doi:10.1186/s41182-025-00688-7)
Supplement: Supplementary file 2 — Supplementary Material 2. [file 41182_2025_688_MOESM2_ESM.docx]

Supplementary Table 1. List of primers used for detection of dengue viruses

| Virus | Gene Name | Orientation | Sequences (5' - 3') | References |
| --- | --- | --- | --- | --- |
| DENV-1 | E-NS1 | Forward | GGA-CTG-CGT-ATG-GAG-TTT-TG | Mason P, et al. (1987) |
|  |  | Reverse | ATG-GGT-TGT-GGC-CTA-ATC-AT | Morita K, et al. (1991) |
| DENV-2 | E | Forward | GTT-CCT-CTG-CAA-ACA-CTC-CA | Deubel V, et al. (1986) |
|  |  | Reverse | GTG-TTA-TTT-TGA-TTT-CCT-TG | Morita K, et al. (1991) |
| DENV-3 | E-NS1 | Forward | GTG-CTT-ACA-CAG-CCC-TAT-TT | Osatomi K, et al. (1990) |
|  |  | Reverse | TCC-ATT-CTC-CCA-AGC-GCC-TG | Morita K, et al. (1991) |
| DENV-4 | NS2 | Forward | CCA-TTA-TGG-CTG-TGT-TGT-TT | Mackow E, et al. (1987) |
|  |  | Reverse | CTT-CAT-CCT-GCT-TCA-CTT-CT | Zhao B, et al. (1986) |
